# Effect of Ultraviolet Activation on Sub-ppm NO_2_ Sensing Dynamics of Poly(3-hexylthiophene)-Bearing Graft Copolymers

**DOI:** 10.3390/s22249824

**Published:** 2022-12-14

**Authors:** Piotr Kaluzynski, Kinga Kepska, Monika Maciuch, Erwin Maciak, Agnieszka Stolarczyk, Marcin Procek, Tomasz Jarosz

**Affiliations:** 1 Department of Optoelectronics, Silesian University of Technology, 2 Krzywoustego Str., 44-100 Gliwice, Poland; 2 Department of Physical Chemistry and Technology of Polymers, Silesian University of Technology, 44-100 Gliwice, Poland

**Keywords:** NOx sensor, ppb, room temperature, poly(3-hexylthiophene), copolymer, ultraviolet activation

## Abstract

Nitrogen dioxide (NO_2_) sensors utilising graft copolymers bearing poly(3-hexylthiophene) chains have been developed and investigated in terms of their operation parameters using different carrier gases (N_2_ or air) and in either dark conditions or with ultraviolet (UV) irradiation. Interestingly, sensor performance improved upon transition from N_2_ to air, with the inverse being true for most NO_2_ sensors. UV irradiation both improved sensor dynamics and stabilised the sensor electrical baseline, allowing sensors based on SilPEG to fulfil the requirements of sensing solutions used in industry (below 10% baseline drift after sensors reach saturation) and making them promising candidates for further development and applications. Based on conducted multi-variate experiments, an initial mechanism underlying the interplay of exposure to oxygen (present in air) and UV irradiation was postulated.

## 1. Introduction

Nitrogen dioxide (NO_2_) is an oxidising gas and a precursor of nitric acid. Nitrogen dioxide is an unfortunately frequent contaminant of air, both at industrial sites and in residential zones. NO_2_ has been found to cause adverse effects to human health [1,2,3]. This contaminant is commonly present in numerous types of industrial [4] and vehicular [5,6] exhaust gases. Civilian and military use of explosives, although less common, is also relevant as both a source of NO_2_ emissions and as a source of the exposure of personnel to this contaminant [7,8].

In light of the hazardous nature of NO_2_, numerous countries worldwide have implemented standards on the maximum acceptable levels of this pollutant in ambient air [9], although it should be noted that these acceptable levels differ both from country to country and between civilian and military regulations [8]. Consequently, detection of NO_2_ is an ongoing research issue, relevant both to industrial applications and air quality monitoring. NO_2_ concentration thresholds as low as 200 ppb have been proposed by the American Conference of Governmental Industrial Hygienists and included in various regulations [10]. Consequently, in order to fulfil such monitoring needs, NO_2_ sensors should have limit of detection (LOD) values of no more than 200 ppb at room temperature, but lower LOD values are preferable.

This criterion is either fulfilled or significantly exceeded by many of the recently reported NO_2_ sensors (Table 1). Although these sensors show LOD values as low as 2.2 ppb NO_2_ in air, their dynamics are limited in many cases. This feature will pose issues with real-time detection applications, particularly in regard to sensor recovery times, which are often manyfold greater than the relevant response times.

In our previous work [11], a comprehensive study of NO_2_ sensing properties of different graft co-polymers containing regioregular poly(3-hexylthiophene) (rr-P3HT) was presented. The investigation was conducted for NO_2_ concentrations in the low ppm range (1–20 ppm) in nitrogen (carrier gas) and at multiple temperatures, from room temperature (RT) up to 100 °C. The noticeable problem shown in the mentioned work was poor stability and a strong baseline drift at RT. This issue was resolved by elevating the temperature of the utilised sensor, resulting in a stable performance. In this context, the use of ultraviolet (UV) irradiation is another means of accelerating the kinetics of the sensors at RT, both for inorganic and organic materials, as shown in our earlier works [12,13]. The use of UV irradiation, however, is dependent on the ability of the material to absorb the radiant energy and to relax the excess energy so as to minimise the UV-induced degradation of the material, particularly in the case of conjugated polymers [14].

In this work, we have investigated the effect of UV irradiation of rrP3HT-bearing graft copolymers on their performance and the improvement in their sensing dynamics in air. Compared to the previous work, the introduced structural modifications of the copolymers not only achieve better usage of the radiant energy, but also allow considerably lower NO_2_ concentrations—in the ppb range—to be detected.

## 2. Materials and Methods

### 2.1. Synthesis of Copolymers

**Figure 1:**
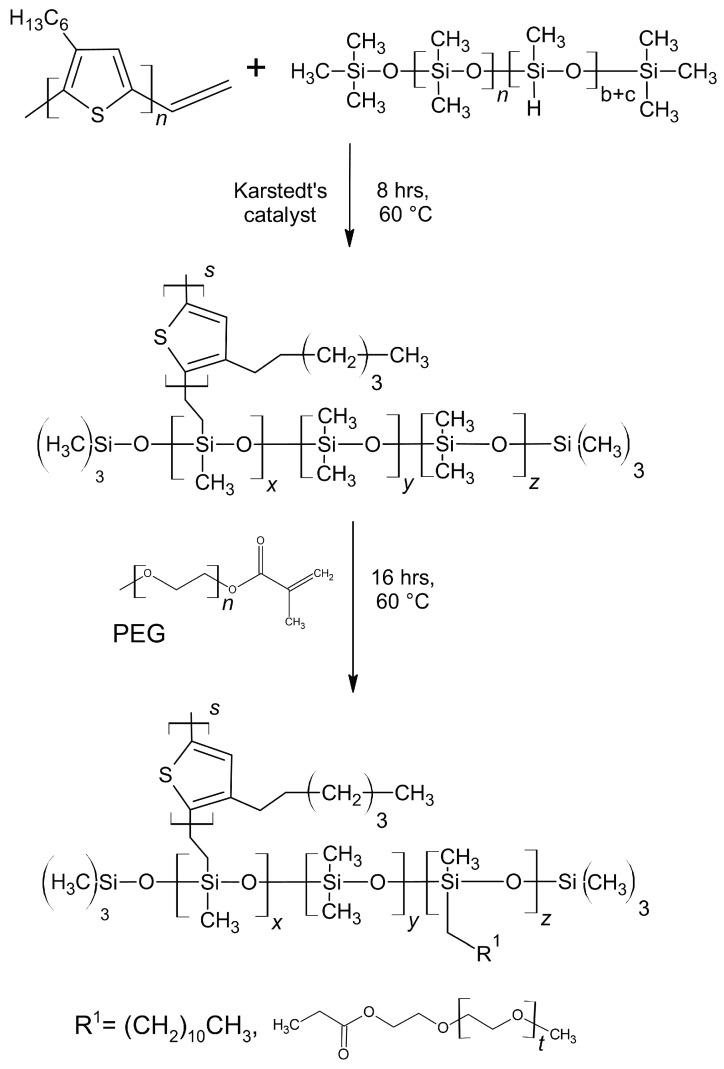
Scheme of the poly(3-hexylthiophenes) (rrP3HT) graft copolymer synthesis route; DodecSIL - R^1^= dodec-1-en, SilPEG - R^1^= PEG.

The synthesis of the vinyl-terminated regioregular poly(3-hexylthiophene) (vin-rrP3HT) via the Grignard metathesis method was conducted based on the procedure described in the literature [30]. All of the reagents were purchased from Sigma Aldrich, Saint Louis, MO, USA. The average molecular weight (M_N_) of vin-rrP3HT was approximately 10,000 g/mol, as determined by the gel permeation chromatography. The investigated graft copolymers were obtained using the method described in the patent application [31] The synthesis was based on the grafting of the vinyl terminated rrP3HT with a poly(ethylene glycol) (PEG) methyl ether methacrylate (M_N_ 950 g/mol) or dodec-1-en onto poly(dimethylsiloxane-co-methylhydrosiloxane), trimethylsilyl terminated) M_N_ 950 g/mol (poly(DMS-co-MHS)) chains (Sigma Aldrich, Saint Louis, MO, USA). Receiving accordingly (poly(DMS)-co-[poly(MHS)-graft-2-vinyl-poly(3-hexylthiophene)]-co-[poly(MHS)-graft-PEG]) (**SilPEG**) and (Poly(DMS)-co-[poly(MHS)-graft-2-vinyl-poly(3-hexylthiophene)]-co-[poly(MHS)-graft-dodec-1-en] (**DodecSIL**). The scheme of the synthesis of the obtained rrP3HT graft copolymers is presented in Figure 1.

The chemical structure of the obtained materials was confirmed by Fourier transform infrared–attenuated total reflectance mode spectroscopy (FT IR ATR) and proton nuclear magnetic resonance spectroscopy (^1^H NMR spectroscopy). The characteristic signals are summarised in the Appendix A (Table A1).

### 2.2. Structural Identification

^1^H NMR analyses were performed in CDCl_3_, on a Varian Unity Inova (Palo Alto, CA, USA) spectrometer with a resonance frequency of 300 MHz using tetramethylsilane (TMS) as an internal standard. FT IR spectroscopy was carried out on a Perkin–Elmer Spectrum Two (Waltham, MA, USA) spectrometer with a UATR (single-reflection diamond) module. The average molecular weights were determined for tetrahydrofuran solutions of sample concentration 1 mg/cm^3^ using a size-exclusion chromatography (SEC) 1100 Agilent (Santa Clara, CA, USA) 1260 Infinity isocratic chromatograph with a differential refractometric MDS RI Detector (USA), calibrated by linear polystyrene standards (580–300,000 g/mol).

### 2.3. UV-Vis-NIR Spectroelectrochemistry

Ultraviolet–visible–near-infrared (UV-Vis-NIR) spectra were registered for polymer films deposited via spin-coating with a typical spectrum acquisition time of 25 ms and with 40 individual spectra being averaged to improve the signal-to-noise ratio. UV-Vis-NIR spectra were recorded using a diode array detector spectrometer set supplied by Ocean Optics that consisted of two measurement units, QE65000 and NIRQuest 512 (responsible for acquiring spectra in the UV-Vis and NIR ranges, respectively) and a DH-2000-BAL balanced deuterium and halogen light source.

The potential was applied using step chronoamperometry, with the sample initially being conditioned by the potential used in the previous step (the minimum applied potential, in the case of the first potential step) for 5 s, following which the potential used in the current step was applied. Conditioning with this second potential was performed for approximately 100 s in order to ensure that all processes taking place in the sample were at equilibrium. Following that, the spectrum for the given applied potential was recorded. A Metrohm Autolab PGSTAT302N potentiostat was used for the purpose of applying the potential staircase to the experimental cell.

### 2.4. Sensing Layers Fabrication

In order to carry out gas measurements, conductive polymer thin film sensor structures on interdigital transducers (IDT) were fabricated. The IDTs were created on a 3×3 mm silicon substrate with a 200 nm thermal SiO_2_ dielectric layer. A photolithography process and physical vapour deposition were used to create electrodes on the surface of the transducer. Firstly, 20 nm of chromium used as an adhesive layer was evaporated, which was covered with a 100 nm layer of gold. The width of the electrode was 3 μm, the distance between the fingers was 10 μm, and the length of the channel was 1500 μm. Thin-film conductive polymer layers were created with the drop coating method. A total of 2.5 mg of conductive polymer material was dissolved in 1 mL of chlorobenzene (POCH, Gliwice, Poland) using 30 min of ultrasonic stirring. The resulting solution was applied to the IDT with the drop coating method using a micropipette (PZ HTL S.A. Lab Solutions, Poland), applying 6 μL of the solution to the substrate for the given type of conductive polymer. Elaborated structures were left to dry completely under a laminar chamber for 12 h on the heater plate and purged with pure nitrogen gas to remove non-adhering materials. Elaborated structures were placed on an Al_2_O_3_ electric heater and connected to chip feedthroughs via ultrasonic bonding, using a 25 μm gold wire. The bonding process was realized using a 53XX-BDA, F& K DELVOTEC wire bonder. The morphology and roughness of the obtained thin films were investigated using atomic force microscopy (AFM) NTEGRA Prima (NT-MDT, Amsterdam, The Netherlands). Topographic imaging was performed in semicontact mode using an HA_NC ETALON cantilever from NT-MDT (255.908 kHz; 12 ± 20% N/m; cantilever length 94 ± 2 μm, width 34 ± 3 μm, RH: 53%, temperature 21 °C). Simultaneously with topographic images, the AFM phase shift was recorded.

### 2.5. Electrical Characterization and Gas Sensing Measurements

Fabricated structures were placed in a specially designed gas measuring chamber equipped with a through-hole technology (THT)-type chip to conduct gas tests in different gaseous atmospheres. Resistance measurements of the sensor’s active polymer layers were conducted using a multichannel switch unit 34970A (Agilent, Santa Clara, CA, USA), whose measurement range was set to 100 MΩ, in which a 500 nA current source is used. Temperature (23.5 °C) was measured by proportional–integral–derivative (PID) controller SR94 (Shimaden, Tokyo, Japan) using a Pt100 sensor attached to an Al_2_O_3_ heater. Measurements were carried out at room temperature, both in dark conditions and under constant UV irradiation (LED, λ = 393 nm confirmed using spectrophotometer HR2000+ES, Ocean Optics, Orlando, FL, USA) through quartz windows, with which the measurement chamber was equipped. Power density of the UV light illuminating the sensors was measured using UV intensity matter model 1000 (SÜSS MicroTec, Garching bei München, Germany) to be 0.2 mW/cm^2^.

The acquisition of measurement data and the preparation of the gas mixtures were performed by a gas mixing server, utilising mass flow controllers (Bronkhorst, HIGH-TECH B.V., The Netherlands) controlled by LabVIEW environment, which allows for precise preparation of gaseous atmospheres. During the measurement process, the relative humidity (RH) of the gas mixture was measured by a RH meter. The RH was maintained at a level of 3.5% ± 1% (at 23.5 °C).

The gas detection measurements were divided according to the type of the used carrier gas (nitrogen and synthetic air) and also according to the conditions of stimulation of the sensor structures with UV radiation (dark conditions and UV-irradiated structures, respectively). One measurement cycle consisted of alternation between exposing the sensor structures to the mixture of a carrier gas and the tested gas (the mixture was prepared so as to have the specified concentration of the tested gas) for 30 min and purging the sensor with the carrier gas for 30 min. The following doses of nitrogen dioxide gas were used: 50, 100, 200, 300, 400, 500, and 1000 ppb of NO_2_ (from gas cylinder with standard mixture of NO_2_ 1000 ppb in N_2_) for each nitrogen dioxide measurement cycle. The sensors were also exposed to other gases (e.g., CO_2_ and NH_3_), in order to determine cross-sensitivity of the sensors. In this case, 10% CO_2_ and 10 ppm NH_3_ an RH 50% @ RT were ran in the measurement cycle, respectively. Regardless of the utilised carrier gas or gas mixture, a constant volumetric gas flow of 400 mL/min was maintained throughout all of the experiments. The short-term baseline drift values were calculated as the values of the response after 30 min of sensor recovery (average from last 20 s); thus, they show the difference between the response value after a recovery cycle and the initial baseline (response equal 0%).

The sensor response for reducing and neutral gases was calculated by using the Formula (1):

(1) $$Response=\frac{\left(R_{g}-R_{a}\right)}{R_{a}}\times 100\%$$

where:

- *R_g_* is the resistance measured in the target gas (e.g., NH_3_, CO_2_, and RH).
- *R_a_* is the resistance measured in carrier gas (baseline value).

For oxidising gases, the sensor response (R) was calculated using Formula (2):

(2) $$Response=(\frac{R_{a}}{R_{g}}-1)\times 100\%$$

where:

- *R_a_* is the resistance measured resistance in carrier gas (baseline value).
- *R_g_* is the resistance measured in target gas (e.g., NO_2_).

The response time (t_resp90%_) was calculated as the time taken to reach 90% of the sensor’s maximum response value. The recovery time (t_rec10%_) was calculated as the time from start of clean carrier gas flow to reach a level of 10% of the maximum response value.

## 3. Results

### 3.1. Microscopic and UV-Vis Spectroscopic Investigations

**Figure 2:**
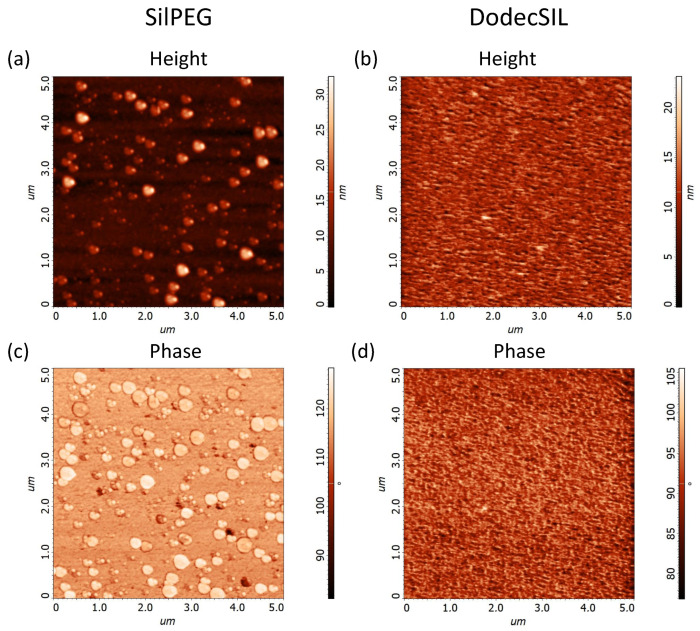
AFM images of polymer films from 5×5 μm area: (**a**) SilPEG height (RMS = 3.7 nm) (**b**) DodecSIL (RMS = 2.3 nm) height; (**c**) SilPEG phase; (**d**) DodecSIL phase.

The morphology of polymer films was examined using AFM (Figure 2). The results show that SilPEG (Figure 2a,c) film contains uniformly distributed circular islands with heights in the range of 10–30 nm and diameters from 50 nm to 400 nm. These islands are probably agglomerates of rrP3HT [32,33]. DodecSIL (Figure 2b,d) exhibits a more uniform non-porous structure with an average of 10 nm (± 2.5 nm) height variation. The root mean square height (RMS) values for 5×5 μm areas of the structures were equal to 3.7 ± 0.1 nm and 2.3 ± 0.1 nm for SilPEG and DodeSIL, respectively [34,35]. This shows that the morphology of SilPEG is more developed than that of DodecSILs. Differences between polymer morphologies depend on the nature of the side chain (PEG or DODEC-1-en). PEG acts simultaneously as a plasticizer and a polyelectrolyte. Hence, it also shows low chain stiffness in comparison to Dodec-1-en (plasticizer). This is why the mobility of rrP3HT chains and crystallization ability are higher in SilPEG than in DodecSIL.

**Figure 3:**
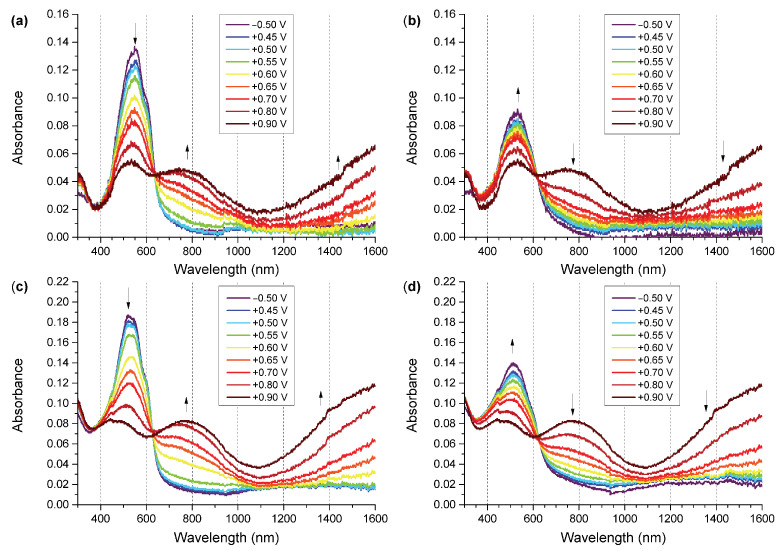
UV-Vis-NIR spectra of polymers during oxidation and reduction process: (**a**) SilPEG oxidation; (**b**) SilPEG reduction; (**c**) DodecSIL oxidation; (**d**) DodecSIL reduction.

The wavelength of light for structures activation has been chosen to ensure stable absorption of polymers regardless of their doping state based on the acquired UV-Vis spectroelectrochemical investigation results (Figure 3). The absorbance of a polymer is a function of its doping level, with ground-state and doped-state polymer segments giving rise to various absorption signals at different wavelengths. In order to avoid the issue of the polymer absorbing different amounts of UV radiation once its doping level changes (due to interactions with the analyte or due to photoexcitation), we have selected a wavelength range of approximately 350–400 nm for light activation, as the absorbance of the polymer in this range does not change noticeably with changes in the doping level of the polymer (Figure 3). It is a well-known problem that UV light leads to photodegradation of rrP3HT [36,37]. This is why the wavelength should be selected to minimise photodegradation and maintain the ability to reliably photogenerate charge carriers. Thus, an illumination wavelength of 393 nm (from an LED source) was chosen for photoactivation of polymers.

### 3.2. Gas Sensing Results

**Figure 4:**
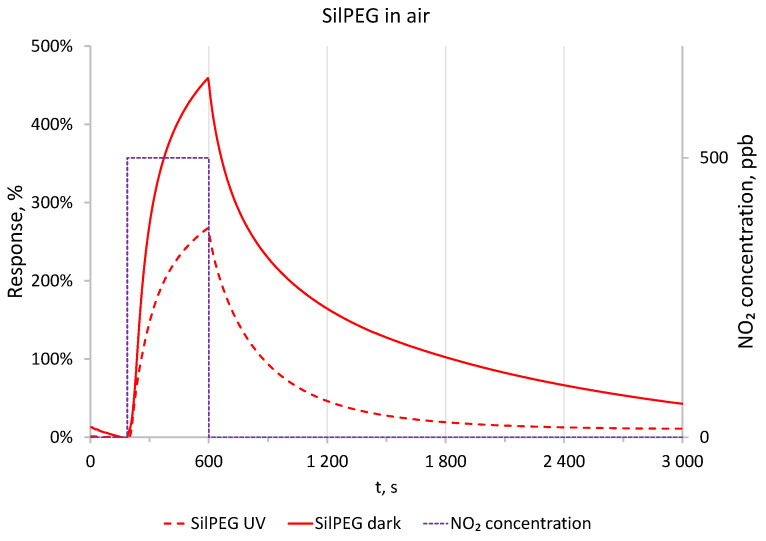
Comparison of responses to 500 ppb of NO_2_ in dark conditions and with UV in air at RT.

The significant recovery time for polythiophene-based sensing structures exposed to NO_2_ is a fundamental issue and key aspect of this work. To show the dynamic behaviour of the sensing structures, the freshly fabricated SilPEG sensing structure was exposed to a NO_2_ concentration of 500 ppb in synthetic air for 400 s followed by purging with the carrier gas (synthetic air) for 2400 s. Tests were conducted in dark conditions and under UV irradiation (Figure 4). The obtained results show that irradiation of the sensing structure with UV enhances the desorption process of the adsorbed gas, thus reducing the recovery time of the sensing structure. In dark conditions, after a recovery period five times longer than the period of exposure to the analyte (NO_2_), the sensing structure still does not achieve a stable response value below 10% of the maximum response value. Under activation by UV, a stable signal was achieved after less than 20 min. The response of the sensing structure after 400 s did not stabilise in either dark conditions or under UV irradiation. Similar results were obtained for the DodecSIL-sensing structure. Based on these results, we selected 30 min to be the duration for exposure and recovery cycles, as this period is sufficient to present the sensing characteristics of the investigated materials.

#### 3.2.1. Nitrogen Conditions

**Figure 5:**
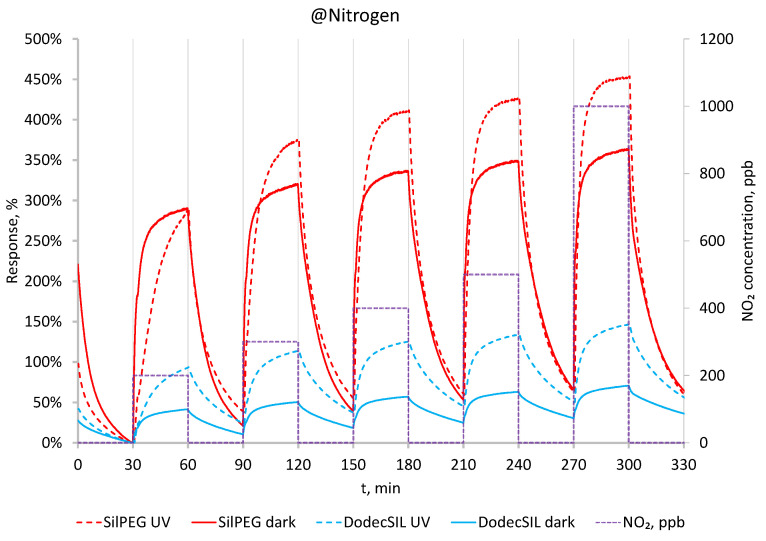
Response to 200–1000 ppb in dark conditions and with UV in N_2_ at RT.

Having obtain the time conditions for gassing of the sensor structure in the first stage, the gas sensing experiments were performed in an oxygen-free atmosphere (in a N_2_ carrier gas). In this case, the polymer films had very high base resistances (exceeding 100 MΩ) and were out of range for the standard measurement unit used. The resistance in N_2_ at dark conditions was examined using Keithley 4200-SCS parameter analyser, and its value was around 250 MΩ for SilPEG. NO_2_, being a strongly oxidising gas, doped the polymer, resulting in its electrical resistance value decreasing to less than 100 MΩ after exposure to 100 ppb for 30 min. The response of sensors using the two copolymers to NO_2_ concentrations in the range of 200–1000 ppb is shown in (Figure 5) with the solid lines. Based on the observed result, SilPEG is much more sensitive to NO_2_ than DodecSIL, which is in line with the results of our previous investigations [11].

The same sensing experiments were repeated under UV light activation (Figure 5 dashed lines). In the N_2_ atmosphere, activation by UV light increases the sensitivity to NO_2_ for both polymers but decreases the response time and does not result in any noticeable improvement in the regeneration of the sensors or in minimising baseline drift.

#### 3.2.2. Air Conditions

The aim of our study was to move from a N_2_ atmosphere to the operation of sensors in the more realistic air atmosphere so as to investigate the utility of using such polymers in sensors operating in real conditions for sub-ppm NO_2_ concentrations. The transition to an air atmosphere often poses a challenge to polymer-based NO_2_ sensors, due to the presence of oxygen, which is also an oxidising agent.

**Figure 6:**
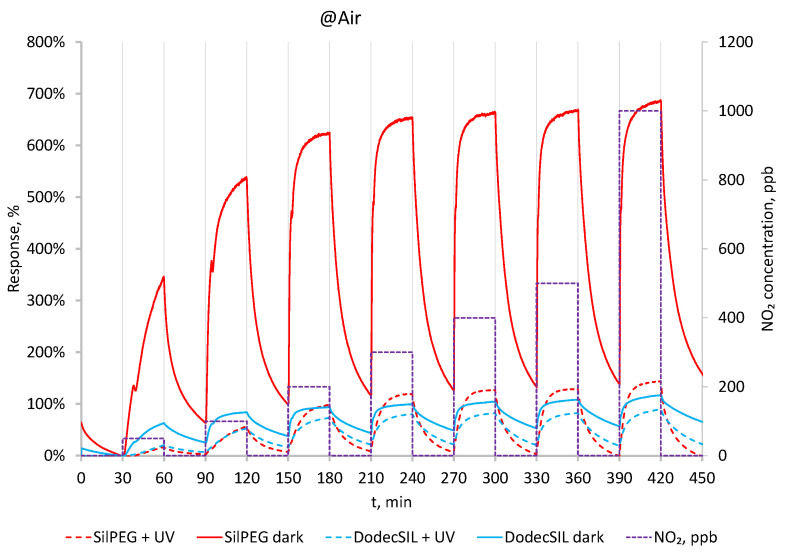
Response to 50–1000 ppb NO_2_ in dark conditions and with UV in air at RT.

In Figure 6, dynamic responses of the polymers to NO_2_ in the range 50–1000 ppb are presented. Solid line represents the data obtained in dark conditions. SilPEGs responses in those conditions are relatively high (340% @ 50 ppb–660% @ 1000 ppb) and are much higher than DodecSILs ones. For both polymers, responses to NO_2_ in air (dark) are higher than its responses in N_2_. This is due to the additive effect of oxidation by NO_2_ and O_2_. When UV activation is applied (Figure 6 dashed lines), the sensitivity of the sensors decreases; however, a significant improvement in baseline stability is observed, even in the relatively low concentration range of 50–100 ppb.

#### 3.2.3. Sensing Parameters

**Figure 7:**
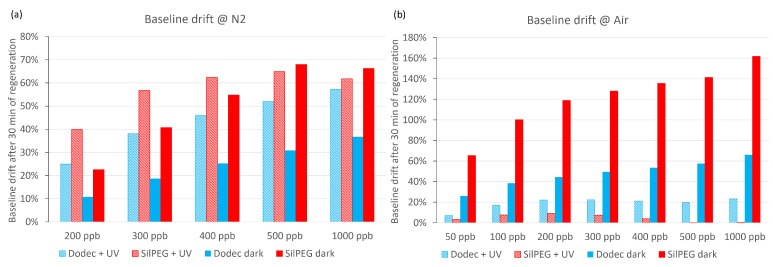
Baseline drifts: (**a**) at nitrogen conditions; (**b**) at air conditions.

The baseline drift of the sensors is a very important issue. In Figure 7, the short-time baseline drifts of sensors after 30 min regeneration in carrier gases (after each examined NO_2_ concentration) are presented. In nitrogen carrier gas (Figure 7a), the drifts exceed 10% in all cases and are comparable in both dark and UV conditions for both polymers. The drift values are proportional to the concentration of NO_2_, which is the result of incomplete regeneration of the sensors after each single gas dosing cycle. There is no significant improvement in baseline stabilisation, and for DodecSIL, the baseline drift even increased when UV activation was applied.

**Figure 8:**
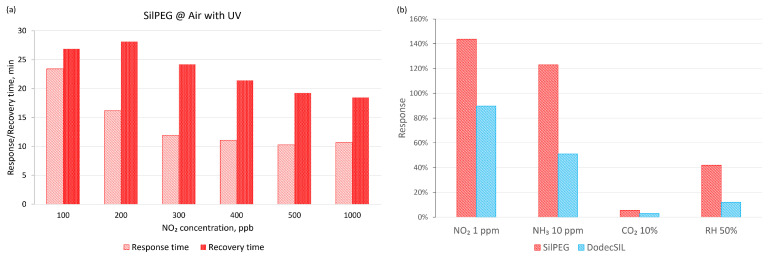
(**a**) Response and recovery times for SilPEG in air with UV; (**b**) selectivity of the polymers in air conditions with UV activation.

When air was used as the carrier gas (Figure 7b), the baseline drift significantly increased in dark conditions in comparison to experiments using nitrogen as the carrier gas. The application of UV activation in air sharply decreases the baseline drift in comparison to any other investigated conditions for both polymers. It is most noticeable in the case of SilPEG, where the baseline returns to a value which does not exceed 10% of the initial resistance for each investigated concentration. Compared to SilPEG, DodecSIL shows about 20% baseline drift for administered concentrations of NO_2_ (200–1000 ppb). In such a situation, we can only consider the regeneration process in the case of SilPEG in air atmosphere with UV; therefore, the analysis of the response time and the recovery time was carried out only for SilPEG in air with UV, which showed the highest response and stability (Figure 8a).

Selectivity was also determined for the interaction of sensors with other common gas analytes, including reducing (NH_3_) and neutral (CO_2_) gases (Figure 8b). The influence of relative humidity (50%) itself on the sensor response was also investigated. The obtained results show a selectivity for NO_2_ (over 144% to 1 ppm) relative to that of NH_3_ (over 123% to 10 ppm). SilPEG for all tested gases showed a higher sensitivity than DodecSIL.

#### 3.2.4. Influence of UV Irradiation

The impact of illuminating the sensor structure of SilPEG with UV light was measured using a Keithley 4200A-SCS parametric analyser with a 1 V DC bias. The measurement cycle was designed to provide more information on the interplay of effects caused by exposure to air and irradiation with UV.

**Figure 9:**
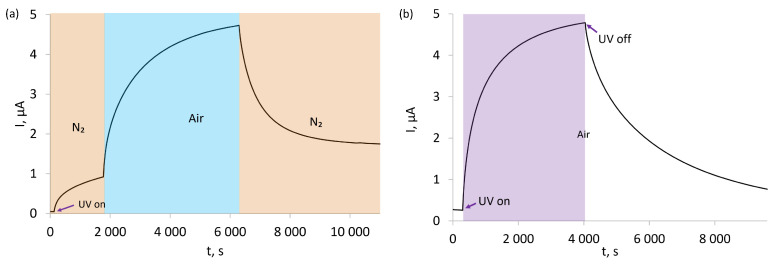
(**a**) Effect of UV activation in nitrogen and effect of exposure to air in UV after N_2_ for SilPEG; (**b**) effect of UV activation in air and relaxation after UV in air for SilPEG.

In the first experiment (Figure 9a), the sensors were subjected to continuous UV irradiation while being immersed in pure N_2_ atmosphere. This irradiation leads to the photogeneration of charge carriers, which is observed as an increasing current flowing through the sensing structure. The current increase is initially fairly rapid, but quickly slows down as the concentration of photogenerated charge carriers becomes sufficient for their recombination to become significant. Once the N_2_ atmosphere is replaced with air, another similarly shaped current increase is observed, but with a much greater magnitude than for the N_2_ atmosphere. Once air is replaced with N_2_,this increased current quickly decays and gradually stabilises at an intermediate level.

The second experiment was conducted entirely in air and was set to examine the influence of UV irradiation on the electrical conductivity of the sensor (Figure 9b). It should be noted that even prior to UV irradiation, the sensor’s baseline electrical resistance was much lower than for the sensor immersed in N_2_, as seen by the much higher currents flowing through it in air than in N_2_. Once UV irradiation was started, the current flowing through the sensor increased almost identically to the case of the first experiment. This current quickly diminished when UV irradiation was ceased.

#### 3.2.5. Stability Studies

**Figure 10:**
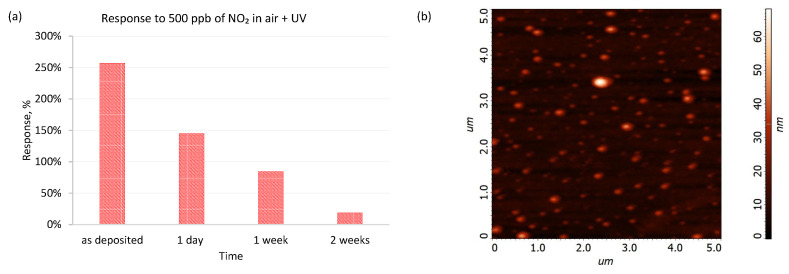
(**a**) Response of SilPEG to 500 ppb of NO_2_ in air under long-term continuous UV illumination; (**b**) AFM image of SilPEG after two weeks aging under UV in air.

Continuous illumination of the sensing structures placed in a flow of in synthetic air with UV resulted in their gradual deactivation. This deactivation is apparent when considering the changes in the response of the sensing structures to 500 ppb NO_2_ in synthetic air carrier gas over time. The response of the “as deposited” sensing structure after 1 h of illumination was 260% but decayed to 145% and 18.5% after one day and two weeks, respectively (Figure 10a). After that period, the sensing structure was investigated using AFM (Figure 10b). There is a noticeable change in the morphology in comparison to the fresh sensing structure (Figure 2a). After ageing, the agglomerates of SilPEG are higher (20–60 nm) and the RMS increased to 4.9 ± 0.1 nm. The decreasing structure sensitivity is accompanied by changes in the morphology of the receptor layer, indicating that long-term exposure to near-UV irradiation induces deactivation of the receptor layer. Although such deactivation can be mitigated by employing UV activation intermittently only during measurement readouts, this would preclude the receptor material from being employed in a continuously operating sensor. Consequently, further work on this receptor material should focus on preventing this deactivation, e.g., via blending the polymer receptor material with more UV-resistant species that could accept the excess radiant energy, such as metal oxides.

## 4. Discussion

### 4.1. UV-Vis-NIR Spectroelectrochemistry

The two types of copolymers show similar UV-Vis absorption features, with the undoped polymer signals being observed at 520 and 546 nm for DodecSil and at 552 nm for SilPEG, corresponding to a slightly lower band gap for SilPEG than for DodecSil. Upon application of increasing potentials, the two polymers behave similarly, exhibiting electrochromic behaviour typical for regioregular poly(3-hexylthiophene), which is the key electroactive component of both copolymers. Upon oxidation, absorption signals corresponding to doped polymer segments begin evolving, centred at approximately 750–770 nm, as well as at lower energies, with only an onset of the signal being observed.

Once the applied potentials are gradually decreased, the doped polymer signals begin decaying and the ground state (undoped) polymer absorption begins recovering. This recovery, however, is only partial, indicative of limited doping state reversibility, with the achievement of high doping levels possibly being tied to degradation of the conjugated system as no doped polymer signals remain after −0.5 V is applied as the final conditioning potential. This doping/dedoping irreversibility is slightly less pronounced for DodecSil (25% loss of initial undoped polymer absorbance) than for SilPEG (34% loss of initial undoped polymer absorbance). Because the interactions of the copolymers with nitrogen dioxide are likely to cause repeated doping and dedoping, DodecSil is likely to exhibit longer lifetimes in NO_2_ sensor configurations, but may have slightly lower responses than SilPEG, likely due to the fact that it undergoes dedoping more rapidly.

The above may also serve to explain the differing effects of UV irradiation on sensor dynamics in nitrogen and in air, as oxygen can interact with spin-bearing (i.e., cation radicals) charge carriers present in doped conjugated polymers, whereas nitrogen does not form such interactions. Upon irradiation, the oxygen–charge carrier interactions may be disturbed, possibly leading to a decomposition of the charge carrier and a return of the polymer segment to the ground undoped state.

### 4.2. Gas Sensing Properties

In general, the response of SilPEG to NO_2_ is higher than that of DodecSIL, regardless of N_2_/air atmosphere or the presence/absence of UV irradiation.

In sensing experiments, the dual UV/air activation mechanism translates into a drastically lowered base resistance (R_a_) of the sensors. On the one hand, this lowered base resistance helps control baseline drift (Figure 7). On the other hand, because NO_2_ is an oxidising gas, a lower base resistance of the sensor translates into a lower achievable response of the sensors to the analyte, as per Equation (2).

### 4.3. Sensing Structure Activation and Operating Mechanism

#### 4.3.1. Activation Mechanism

Interestingly, the effects of both UV and air exposure are not entirely straightforward, as in the case of these poly(3-alkylthiophene)-based receptor layers, an interplay of the two factors is observed (Figure 9), even prior to the sensors being exposed to the analyte (NO_2_). The observed activation of the receptor layers in air upon exposure to UV irradiation is tied to the photogeneration of charge carriers on the polymer chain. This photogeneration takes place via the absorption of a quantum of energy (h*ν*) to induce charge separation on neighbouring repeat units of the polymer (Th) and is a well-known phenomenon [38]. If the specific nature of the charge carriers is not considered, the process can be simplified as:

$$2\mathrm{Th}\overset{h\nu }{⇌}{\mathrm{Th}}^{-\cdot }+{\mathrm{Th}}^{+\cdot }$$

It should be noted that photogeneration of charge carriers is accompanied by an opposing process: recombination of charge carriers. At steady conditions, the two processes eventually achieve equilibrium, resulting in the observed, gradually decelerating increase in current flowing through the sensing structures (Figure 9) when they are irradiated while being immersed in a flow of nitrogen.

Polythiophenes are p-type conductors, that is, their conductivity is realised primarily via the movement of positive charges which are, in this case, expressed as radical cations (${\mathrm{Th}}^{+\cdot }$) that can be formed either through redox doping or through photo-induced charge separation, as in the above process. The negatively charged radical anions (${\mathrm{Th}}^{-\cdot }$) produced in this process exhibit low charge-carrier mobility.

Investigation of the activation of our sensing structures by UV and exposure to air (Figure 9) showed that a combination of UV and air exposure leads to a significant increase in current and, therefore, a significant rise in the conductivity of the tested polymers. Because the conductivity of polythiophenes is tied to the population of positively charged charge carriers, an increase in conductivity must originate from an increase in the charge-carrier population.

The charge-carrier population is tied to the equilibrium of the above reaction, which, in the simplest case, is a function of the intensity of UV irradiation (formation of charge carriers) and the recombination of positive and negative charge carriers (charge-carrier decay). The increase in current observed upon exposing the sensing structures to air results from a significantly higher charge-carrier population, indicating that a new photogeneration/recombination equilibrium state being established in the polymer. Because during the aforementioned experiments the irradiation intensity was kept constant, the rate of charge-carrier recombination must have been decreased once the sensing structures were exposed to air.

**Figure 11:**
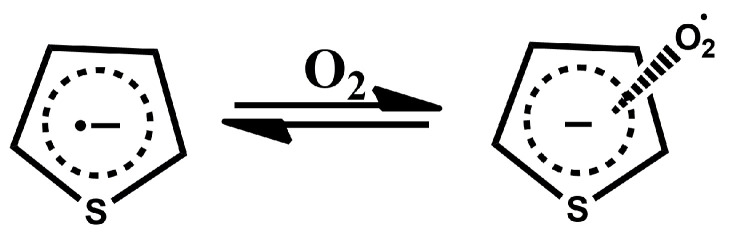
Thiophene–oxygen charge-transfer complex, analogous to the charge-transfer complex discovered for non-doped polythiophenes [39].

The reason for this shift in equilibrium can be found in the fact that the formation of charge-transfer complexes between even undoped polythiophenes and oxygen is already a well-known phenomenon [39]. The formation of these charge-transfer complexes is based on the transfer of electrons from polythiophenes to O_2_, resulting in the formation of a radical cation on the polymer chain. We hypothesise that in our case, oxygen present in air interacts with the negatively charged repeat units in our polymers as per the following reaction (Figure 11):

$${\mathrm{Th}}^{-\cdot }+O_{2}⇌[\mathrm{Th}-][{O_{2}}^{\cdot }]$$

In the case of photogenerated charge carriers, not only will such interactions have a greater driving force (due to the potential energy gain caused by pairing unpaired electrons constituting the charge carriers and present in O_2_ molecules), but in the case of negative charge carriers, will result in a charge-transfer complex $\left[\mathrm{Th}-][{O_{2}}^{\cdot }\right]$ that has no positive charge on the polymer chain and a negative charge on the strongly electronegative oxygen. This complex is hypothesised to be more stable than Th^−·^. Consequently, such a “bonding” of negatively charged photogenerated charge carriers would hinder recombination while maintaining the presence and relatively high mobility of positively charged charge carriers ${\mathrm{Th}}^{+\cdot }$ This would directly explain the observed experimental results (Figure 5, Figure 6, Figure 7, Figure 8 and Figure 9) and be in line with the conductivity properties of polythiophenes.

#### 4.3.2. Sensing Mechanism

The mechanism underlying the decreasing resistance of polythiophene receptor materials upon exposure to NO_2_, used for detection of this analyte, has been the subject of multiple research efforts. The general consensus is that the oxidising nature of NO_2_ causes an increase in positively charged charge-carrier populations, similar to the case of standard redox doping [24]. This postulated mechanism is corroborated by the fact that exposure oxidising and reducing gases, respectively, causes a decrease and increase in the resistance of the polymers, as would be expected in the case of redox doping. In our experimental work, we have found no evidence to the contrary; hence, the process can be described via the following chemical reaction:

$$\mathrm{Th}+{\mathrm{NO}}_{2}⇌{\mathrm{Th}}^{+\cdot }+{{\mathrm{NO}}_{2}}^{-\cdot }$$

In the case of sensing structures activated by UV, the presence of negatively charged species (${\mathrm{Th}}^{-\cdot }$ for sensing structures immersed in nitrogen and a mixture of ${\mathrm{Th}}^{-\cdot }$ and $\left[\mathrm{Th}-\right]\left[{O_{2}}^{\cdot }\right]$ for sensing structures immersed in air) also needs to be taken into consideration. Because these species are the reduced form of the ground state Th repeat units, they should undergo oxidation by NO_2_ more readily relative to Th itself. In the case of the postulated charge-transfer complex, oxidation would also result in the release of the oxygen molecule from the complex, as follows:

$${\mathrm{Th}}^{-\cdot }+{\mathrm{NO}}_{2}⇌\mathrm{Th}+{{\mathrm{NO}}_{2}}^{-\cdot }$$

$$\left[\mathrm{Th}-\right]\left[{O_{2}}^{\cdot }\right]+{\mathrm{NO}}_{2}⇌\mathrm{Th}+O_{2}+{{\mathrm{NO}}_{2}}^{-\cdot }$$

These processes are expected to drastically reduce the population of the negatively charged charge carriers produced via UV irradiation, effectively preventing charge-carrier recombination and giving rise to a very significant increase in the conductivity of our receptor materials, in line with the observed experimental results (Figure 5 and Figure 6).

## 5. Conclusions

To summarise, not only did UV activation significantly improve the dynamics of operation of the sensing structures in experiments using air as a carrier gas, but it also helped minimise the short-term baseline drift for the sensing structures, particularly for ones based on SilPEG. UV activation for SilPEG allowed for a short-term baseline drift below 10% after the sensing structures reach saturation at a given NO_2_ (analyte) concentration. However, the gradual deactivation of the receptor material in these conditions necessitates either employing an intermittent operating regime of prospective sensors or preventing this deactivation, at least partially, e.g., by introducing additives capable of absorbing excess UV radiation into the polymer receptor material matrix. Metal oxides, such as zinc oxide, are one prospective type of additives that could significantly improve the resistance of the receptor material to UV irradiation and perhaps even improve the performance of the sensing structures. Such functionalization of proposed polymers could also solve the problems with the long-term stability and improve the selectivity and response dynamic so that applications may be found in the automotive and energy industries, as well as in air quality monitoring.

The use of experiments alternating UV activation and N_2_/air carrier gas exchange allowed us to formulate an initial postulate about the processes taking place within those receptor layers. The proposed mechanism explains the obtained experimental results to a better degree and is more in line with the properties of conjugated polymers than the most commonly postulated mechanism based on the features of inorganic semiconductors that involves photo-stimulated desorption of oxygen from the sensing structure [40].

## Figures and Tables

**Table 1:** Recently reported room temperature NO_2_ sensors.

Material	t_resp_/t_rec_	LOD	Response [%] (Concentration)	RH [%]	Ref.
PEGSil ^a^	330 s/>30 min	0.2 ppm	1330 (1 ppm) *	6	[11]
DodecSil ^b^	560 s/>30 min	0.1 ppm	450 (1 ppm) *	6	[11]
PPy/RGO-aryl-COOH ^c^	129 s/114 s	-	30 (2 ppm) †	0	[15]
multilayer rrP3HT:SEBS ^d^	-	2.3 ppb	1053 (30 ppm) †	-	[16]
PBTTT/GO ^e^	75 s/523 s	0.37 ppm	174 (10 ppm) †	54	[17]
TIPS-pentacene/*p*-6P ^f^	180 s/360 s	300 ppb	6300 (5 ppm) ^†^	0	[18]
polyaniline	55/68 s	50 ppb	80 (1 ppm) †	-	[19]
PQT-12 ^g^	41/-	100 ppb	24 (100 ppb) †	56	[20]
T-2DP ^h^	35–47/56–140 s	2.2 ppb	505 (1 ppm) †	-	[21]
H_2_[Pc(OCH_2_(CF_2_)_6_CF_3_)_4_] ^i^	1/9 min	100 ppb	12 (1 ppm) ^†^	-	[22]
polypyrrole	126–218/374–2170 s	-	12 (10 ppm) ^†^	-	[23]
polythiophene	220–297/585–1603 s	-	9 (10 ppm) ^†^	0	[24]
rrP3HT:PVK ^j^	-	139.3 ppb	700 (0.6 ppm) ^†^	-	[25]
PRGO ^k^	10/15 min	20 ppb	89 (500 ppb) ^†^	-	[26]
RGO-PTh ^l^	-	0.52 ppm	2636 (10 ppm) *	-	[27]
CuPc ^m^	4–8/30 min	-	800 (3 ppm) *	-	[28]
Mo_2_TiC_2_T_x_/MoS_2_	60/154 s	2.5 ppb	70 (2 ppm)	50	[29]

* response calculated as $R_{a}/R_{g}$; ${}^{†}$ response calculated as $(R_{a}-R_{g})/R_{a}$ or $(I_{a}-I_{g})/I_{a}$; ^a^ polymethylsiloxane grafted with poly(3-hexylthiophene) and poly(ethylene glycol); ^b^ polymethylsiloxane grafted with poly(3-hexylthiophene) and dodecyl chains; ^c^ polypyrrole/reduced graphene oxide functionalized with aryl 4-carboxybenzene diazonium salt; ^d^ poly(3-hexylthiophene):polystyrene-block-poly(ethylene-*ran*-butylene)-block-polystyrene blend (4:1); ^e^ poly[2,5-bis(3-tetradecylthiophen-2-yl)thieno[3,2-b]thiophene]/graphene oxide nanohybrid composite; ^f^ 6,13-bis(triisopropylsilylethynyl)pentacene/para-sexiphenyl; ^g^ poly(3,3‴-dialkylquaterthiophene); ^h^ triazine-based 2D polymer; ^i^ 2(3),9(10),16(17),23(24)-tetrakis(2,2,3,3,4,4,5,5,6,6,7,7,8,8,8-pentadecafluorooctyloxy)phthalocyanine; ^j^ poly(3-hexylthiophene):poly(vinylcarbazole) blend (1:1); ^k^ porous reduced graphene oxide; ^l^ hybrid of polythiophene with thylenediamine-modified reduced graphene oxide (5%); ^m^ copper(II) phthalocyanine.

## Data Availability

Experimental data sets can be obtained directly from the Authors.

## Abbreviations

The following abbreviations are used in this manuscript:

^1^H NMR	proton nuclear magnetic resonance spectroscopy
AFM	atomic force microscopy
ATR	attenuated total reflectance mode
DodecSIL	poly(dimethylsiloxane)-co-[poly(metylhydrosiloxane)-graft-2-vinyl-
	poly(3 -hexylthiophene)]-co-[poly(metylhydrosiloxane)-graft-dodec-1-en])
FT IR	Fourier transform infrared spectroscopy
IDT	interdigital transducer
LED	light-emitting diode
LOD	limit of detection
M_N_	number average molecular weight
PID	proportional–integral–derivative
ppb	parts per billion
ppm	parts per million
R	sensor response
RH	relative humidity
RMS	root mean square height
rrP3HT	regioregular poly(3-hexylthiophene)
RT	room temperature
SEC	size-exclusion chromatography
SilPEG	poly(dimethylsiloxane)-co-[poly(metylhydrosiloxane)-graft-2-vinyl-
	poly(3-hexylthiophene)]-co-[poly(metylhydrosiloxane)-graft-poly(ethylene glycol]
	methyl ether methacrylate)
THT	through-hole technology
TMS	tetramethylsilane
UV	ultraviolet
UV-Vis-NIR	ultraviolet-visible-near infrared

## Appendix A

**Table A1:** Assignments of the main ^1^H NMR and FT IR signals of SilPEG and DodecSIL.

Copolymer	^1^H NMR signal: ppm (assignment)
SilPEG	0.09 (C**H**_3_-Si-O-); 0.50 (-C**H**_2_-Si-O-), 0.91 (-CH_2_-C**H**_3_); 1.34 (-(C**H**_2_)_3_-); 1.71 (-C**H**_2_-CH_2_-C_Ar_); 2.80 (-C**H**_2_-C_Ar_); 3.48 (-Si-CH_2_-C**H**_2_-C_Ar_); 6.98 (**H**-C_Ar_)
DodecSIL	0.09 (C**H**_3_-Si-O-); 0.91 (-CH_2_-C**H**_3_); 1.36 (-(C**H**_2_)_3_-); 1.71 (-C**H**_2_-CH_2_-C_Ar_); 2.80 (-C**H**_2_-C_Ar_); 3.38 (C**H**_3_-O-); 3.64 (-C**H**_2_-O-); 4.22 (CH_2_-C**H**_2_-C(=O)-); 6.98 (**H**-C_Ar_)
**Copolymer**	**IR signal: ν [cm^−1^] (assignment) ${}^{1}$**
SilPEG	794 [δ^1^ (**Si-(CH_3_)_2_**), s^2^]; 1016 [ν_asym._(**Si-O-Si**), vs]; 1260 [δ_asym._(**Si-CH_3_**), s]; 1375 [δ_asym._(**-CH2-**), w]; 1456 [ν_asym._(**C_Ar_=C_Ar_**), m]; 1511 [ν_sym._(**C_Ar_=C_Ar_**), w]; 2855 [ν_sym._(**C-H**), s]; 2923 [ν_asym._(**C-H**), vs]; 2957 [ν_sym._(**C-H**), s]; 3056 [ν(**C_Ar_-H**), w]
DodecSIL	795 [δ(**Si-(CH_3_)_2_**), s]; 1022 [ν_asym._(**Si-O-Si**), vs]; 1260 [δ_asym._(**Si-CH_3_**), s]; 1379 [δ_sym._(**-CH_2_-**), w]; 1456 [ν_asym._(**C_Ar_=C_Ar_**), m]; 1508 [ν_sym._(**C_Ar_=C_Ar_**), w]; 1735 [ν(**C=O**), w]; 2853 [ν_sym._(**C-H**), s]; 2926 [ν_asym._(**C-H**), vs]; 2952 [ν_sym._(**C-H**), s]; 3055 [ν(**C_Ar_-H**), w]

${}^{1}$ vibration type: ν—stretching; δ—deformation; sym.—symetric; asym.—asymetric; band intensity: w—week; m—medium; s—strong; vs—very strong.
